# Perioperative Management of Pediatric Epilepsy Neuromodulation Devices

**DOI:** 10.3390/children13040517

**Published:** 2026-04-08

**Authors:** Young May Cha, Ashley Smith, Hubert A. Benzon, Becky J. Wong

**Affiliations:** 1 Department of Anesthesiology, Children’s Hospital Colorado, University of Colorado School of Medicine, Aurora, CO 80045, USA; youngmay.cha@childrenscolorado.org; 2 Department of Anesthesiology & Pain Medicine, Nationwide Children’s Hospital, The Ohio State University College of Medicine, Columbus, OH 43205, USA; ashley.smith5@nationwidechildrens.org; 3 Department of Pediatric Anesthesiology, Ann and Robert H. Lurie Children’s Hospital of Chicago, Chicago, IL 60611, USA; hbenzon@luriechildrens.org; 4 Department of Anesthesiology, Perioperative and Pain Medicine, Stanford University School of Medicine, Stanford, CA 94305, USA

**Keywords:** drug-resistant epilepsy, perioperative management, vagus nerve stimulation, deep brain stimulation, responsive neurostimulation

## Abstract

**Highlights:**

**What are the main findings?**

• Neuromodulation devices (VNS, DBS, and RNS) are an increasingly utilized treatment option for pediatric drug-resistant epilepsy (DRE). More pediatric patients are presenting for surgeries related or unrelated to device placement.
• We define what these devices are, how they work, and their efficacy.

**What are the implications of the main findings?**

• Perioperative management of these devices requires device-specific knowledge.
• We discuss device-specific anatomy, potential intraoperative physiological effects from the device, and strategies to protect device leads and generators, with the goal of continued perioperative seizure suppression for DRE.

**Abstract:**

Drug-resistant epilepsy (DRE) has a significant burden on children and their families that extends beyond seizure management. Surgery can be a curative treatment but is sometimes not an option for certain generalized epilepsies or epilepsy in an eloquent region. Neuromodulation therapies (vagus nerve stimulation–VNS, deep brain stimulation–DBS, and responsive neurostimulation–RNS) have emerged as effective palliative treatments to mitigate seizure burden. Only VNS is FDA-approved for use in certain pediatric populations for epilepsy, but all are used off-label to treat pediatric drug-resistant epilepsy. This review provides an overview of these therapies, the perioperative considerations related to their implantation, and the perioperative considerations related to managing a device in situ. Care must be taken to avoid unintentional harm to the device, the leads, and the generator. Procedures must be cognizant of possible physiological changes that can occur intraoperatively and anatomic restrictions due to lead/generator placement. Although there is still a need for more long-term safety data regarding the use of neuromodulation devices in children, the current data demonstrate good efficacy and safety thus far. More children are likely to receive these devices for treatment, and so continued training and education will be needed for health care providers to maintain device longevity and safety.

## 1. Introduction

Roughly 3 out of 10 children with epilepsy will develop drug-resistant epilepsy (DRE) [1,2], which is defined as failure of two antiseizure medication schedules to achieve seizure freedom for 12 months or three times the inter-seizure interval prior to treatment [3]. Onset of epilepsy within the first three years of life has the highest risk for DRE [1,2]. Other risk factors include focal or multiple seizure types, high baseline seizure frequency, abnormal diagnostic testing (e.g., electroencephalography (EEG), neuroimaging, or neurological examination), structural etiology, and psychiatric comorbidity [1]. Certain syndromes carry higher risks as well, such as developmental and epileptic encephalopathies (e.g., West syndrome, Lennox–Gastaut syndrome, and Dravet syndrome). Mortality is also higher and often from complications rather than the epilepsy itself [3].

Neurodevelopmental outcomes are worse in DRE. Patients with DRE have an 11.4 point lower full-scale IQ vs. non-pharmaco-resistant epilepsy [4]. The effect of uncontrolled seizures on cognition decreases with increasing age of seizure onset, suggesting the early brain is particularly vulnerable. When followed into adolescence and young adulthood, 45% of patients had impaired cognition across multiple domains. Improved outcomes were associated with better baseline cognition, older age at epilepsy onset, and improved seizure control at follow-up 4–11 years later [5]. Quality of life is also reduced, with poorer educational outcomes, lower employment rates in adulthood, increased psychiatric comorbidity, and decreased physical functioning. Lower IQ, fewer family resources, and caregiver unemployment all independently predicted a diminished quality of life [6].

Epilepsy surgery can be a curative treatment option for DRE. Some patients may not be eligible for resective surgery due to multifocal epilepsy, generalized epilepsy, or focal epilepsy in an eloquent region. Some patients fail surgery and others decline the option because larger resections come with increased neurocognitive risk. Neuromodulation therapies offer a palliative option to decrease seizure burden [7,8]. This review focuses on surgically implanted neuromodulation devices: vagus nerve stimulation (VNS), deep brain stimulation (DBS), and responsive neurostimulation (RNS) (Table 1). VNS therapy is the oldest neuromodulation therapy and currently the only device FDA-approved for use in children with epilepsy [7]. DBS and RNS are used off-label in pediatric epilepsy, and there is a small but growing literature on their efficacy and safety.

Children who receive neuromodulation therapies undergo multiple anesthetics, not only for device placement, but also for battery replacement, device adjustments, and even routine imaging studies because children generally cannot tolerate being in the magnetic resonance imaging (MRI) scanner awake. Children also have unique airway considerations and different autonomic physiology than adults that affect their perioperative management. Many children with DRE have comorbidities or other genetic abnormalities that can further impact their physiology and elevate anesthetic risk. Given the high prevalence of DRE, the subsequent medical comorbidities, and the significant burden it places on the patients and their families, it is expected that neuromodulation therapies will continue to grow and fill this clinical gap. However, much of the existing data from the literature is from adults, and the neuromodulation devices are being used as off-label in pediatric patients. This narrative review provides a practical overview of neuromodulation therapies and their perioperative considerations.

## 2. Neuromodulation Therapies

### 2.1. Vagus Nerve Stimulation

VNS therapy was first approved for adults and adolescents over 12 years old with focal DRE in 1997. Approval was extended in 2017 for ages 4 years and older with refractory focal epilepsy, generalized epilepsy, and Lennox–Gestaut syndrome [7]. A generator is implanted in the chest to stimulate the left vagus nerve. Stimulation is usually started a few weeks after initial implantation [10]. A magnet passed over the generator prompts additional stimulation during a seizure. VNS devices require no craniotomy and minimal maintenance, other than battery replacement and revision surgery for lead revision/malfunction. Of the three neuromodulation devices, VNS devices have the most pediatric data with decreased seizure rates comparable to the adult population [11,12]. Seizure reduction is commonly measured by the 50% responder rate, which is the proportion of patients who attain at least a 50% reduction in baseline seizure frequency during the specified follow-up period. We report responder rates to illustrate device efficacy, but the variability of responder rates depending on follow-up limit their utility as a direct comparison of efficacy between the devices. VNS therapy has been reported to have a pooled 50% responder rate of 56.4% in a 2021 meta-analysis of 101 pediatric studies [13], and even helped clinical depression [7]. Children who tried fewer medications prior to VNS and children with a later onset of epilepsy were linked to having higher responder rates [13]. VNS therapy has also been associated with an improvement in quality of life with improved concentration, energy, mood, and progress in school.

Efficacy rates in children differ due to variability in VNS titration and dosing practices. Even with high stimulation parameters, devices must be started low and ramped up over time. Seizure reduction seems to improve over time with 50% responder rates approaching 50% at 2 years in a 2014 pediatric multicenter European retrospective study [14]. A more recent retrospective study in 2025 found higher responder rates in children with a high stimulation protocol (as high as 90.5% for focal epilepsy and as high as 89.7% for Lennox–Gastaut syndrome at 4-year follow-up) [15]. Children who started VNS within 2 years of seizure clinical onset and who used a high stimulation, ultra-rapid duty cycling correlated with better seizure control [15]. Typically, VNS stimulates according to a fixed schedule, but newer closed-loop models automatically trigger stimulation after predefined increases in heart rate that could be predictive of a seizure [16]. Complication rates in young children are generally low and include wound infections, hoarseness, mild cough, hyperactivity, and, rarely, dysautonomia [15,17].

### 2.2. Deep Brain Stimulation

DBS therapy uses an open loop system like VNS. The battery and pulse generator are implanted in the chest and extension wires are threaded through the neck to the target brain region. Some DBS models have batteries that are rechargeable. The anterior nucleus of the thalamus is often targeted for focal epilepsy and the centromedian nucleus of the thalamus is often targeted for generalized epilepsy [18]. The hippocampus can be targeted for temporal lobe seizures. DBS received FDA approval for targeting the anterior thalamus in adults with focal DRE after the SANTE trial: a randomized, double-blind controlled trial of adults who failed at least three antiseizure medications, which showed that implantation reduced seizures by 75% at 7 years post-implantation [19]. DBS delivers continuous or cycling stimulation that is thought to reduce interictal spikes by attenuating functional neural networks and/or interfering with synchronization of epileptic networks [7]. Like VNS, a magnet passed over the generator can deliver additional stimulation during a seizure.

Responder rates for DBS are reported to be as high as 75% at 2 years in a systematic review of 35 pediatric studies [18]. After the SANTE trial showed substantial seizure reduction and DBS gained FDA approval for DRE, there have been more data on DBS use to treat pediatric DRE. DBS may offer superior efficacy in the first year after implantation. Compared with RNS and VNS, DBS has the highest reported responder rate for Lennox–Gastaut syndrome at 69.7% in a meta-analysis of 11 studies with both pediatric and adult patients [20]. The ADVANCE trial, a partially randomized patient preference prospective trial, showed add-on DBS resulted in 51.9% seizure reduction versus 12.3% with only continued VNS optimization in children aged 8–17 [21]. Because DBS is FDA approved to treat pediatric dystonia, there are more robust data on pediatric complications, which include infection, hardware failure, and difficulty connecting properly to recharge in models that have a rechargeable battery [22].

### 2.3. Responsive Neurostimulation

RNS therapy is a closed-loop neuromodulation therapy that is implanted intracranially and continuously monitors EEG at the seizure focus and delivers short bursts of high frequency stimulation on detection of ictal intracranial EEG. The internal pulse generator is implanted in the cranium. RNS devices were approved by the FDA for use in adults with intractable focal epilepsy in 2013. Their use has expanded to off-label use in pediatrics and for more generalized seizure disorders. Ideally, RNS targets a well-defined seizure focus identified through imaging, EEG, or intracranial monitoring. Common targets for RNS are the centromedian nucleus of the thalamus and the anterior thalamic nucleus [18,23,24]. Acute stimulation interrupts seizure activity, and chronic use may facilitate long-term neuromodulation that further improves seizure reduction [7,8]. Case studies in children have shown seizure reduction with multifocal epilepsy and generalized epilepsy syndromes, which have been challenging to treat via other modalities.

Responder rates for RNS are higher (66.7–71%) than for VNS in two retrospective single-center reviews which include both pediatric and adult patients [25,26], and rates appear even higher for certain types of epilepsy (81–93%) in a systematic review of eight pediatric studies [27]. Nearly half of patients reported improvements in mood, behavior, sleep, and social interactions [26]. Cognitive domains improve, depending on the brain region being stimulated [7]. Because the device is intracranial, there are significant concerns about the allowable lower age limit. Rapid skull growth in the first 2 years of life can impact device location, and repeated surgeries for adjustments, repair of lead fracture, or battery replacement can be problematic for children who have a thinner scalp [28]. Adverse events in pediatric RNS have been reported as low (the most common is wound infection at <10%) and comparable to adult RNS and pediatric VNS/DBS in a systematic review [27]. The closed-loop design of RNS minimizes stimulation during normal brain activity, which helps mitigate possible cognitive or behavioral side effects in a developing child whose brain may be more susceptible to these effects. Because it is intracranial, it can also provide long-term electrophysiological seizure recordings from the seizure focus.

### 2.4. Combination Therapies

Given the importance of attaining adequate seizure control as quickly as possible, there is growing literature on the use of combination neuromodulation therapies for pediatric DRE. Commonly reported combinations are VNS with either DBS or RNS, often adding the second when VNS alone is insufficient to obtain good seizure control. DBS/RNS can provide a more targeted neuromodulation therapy to specific cortical or subcortical areas on top of the more general modulation from VNS. A case series reported seven patients <21 years old who tolerated a VNS/RNS combination without any device interactions or major adverse events. All seven patients reported a 75–99% reduction in seizure frequency [29]. Choosing which device is best requires complex discussion about the risks and benefits with the patient/caregiver, neurologist and the neurosurgeon.

## 3. Preoperative Evaluation and Planning

Multidisciplinary planning with all members of the pediatric epilepsy program is required for children undergoing surgery for these neuromodulation devices. If a neuromodulation device is already in place, preoperative evaluation should include device interrogation to confirm function, battery life, and document baseline settings. Device cards with product information are provided to families by manufacturers and indicate the device type, MRI compatibility, and instructions to stop stimulation with the magnet. The child’s neurologist or inpatient neurology service may need to be consulted to assist with perioperative device management.

**Figure 1:**
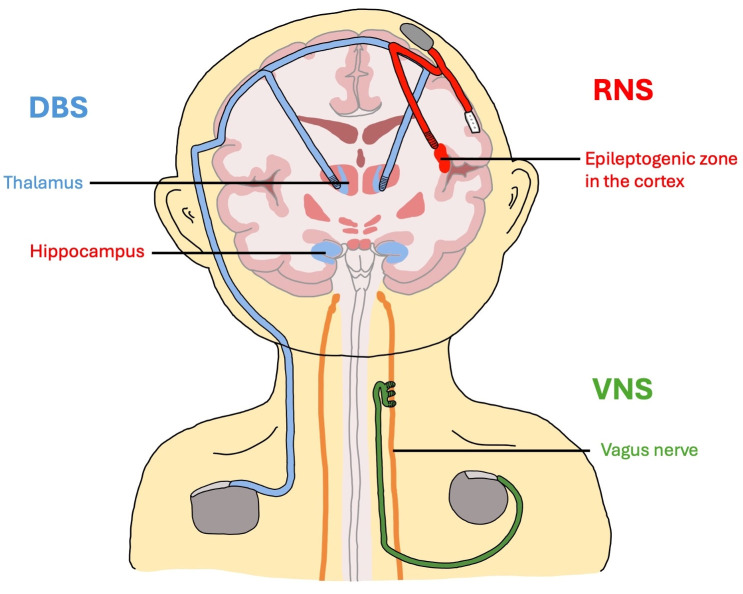
Schematic of placement of neuromodulation devices with lead and generator locations as well as targeted brain parenchyma. VNS targets the vagus nerve. The targets for DBS and RNS can vary. DBS electrodes are commonly placed in the anterior nucleus or centromedian nucleus of the thalamus. RNS electrodes are placed by the neurosurgeon in a location considered to be a likely seizure focus and can target locations in the cortex, thalamus, and hippocampus, for example. Caution is required during central line placement, as VNS and some DBS systems use chest wall pulse generators and tunneling paths that may be at risk of damage or infection with subclavian or internal jugular access. Drawing credit: A. Smith, Y.M. Cha.

The location of the device, leads, and battery should be documented as these components may affect anesthetic or surgical management (Figure 1). Coordination with pediatric neurology to determine appropriate perioperative management of stimulation parameters is important because sometimes devices may need to be adjusted or temporarily disabled to optimize surgical conditions. This need must be balanced with minimizing the risk of breakthrough seizures. Clinicians should also remain vigilant for seizure exacerbation or new stimulation-related side effects in the early postoperative period.

## 4. Medication Management

Patients presenting for neuromodulation device placement are commonly maintained on one or more antiseizure medications, and missing antiseizure medication doses on the day of surgery due to nil per os guidelines can increase the risk of seizures during the perioperative period. Patients should take their antiseizure medications on the day of the procedure to minimize the risk of seizures during the perioperative period. Many medications such as levetiracetam, phenytoin, valproic acid, and newer medications lacosamide and brivaracetam can be converted to intravenous formulations from per os equivalents. However, other antiseizure medications, such as lamotrigine, topiramate, gabapentin, felbamate, and newer medications clobazam and vigabatrin do not have intravenous formulations and may still need to be administered per os [30]. Even with *nil* per os guidelines, patients are generally permitted essential medications with a sip of water prior to surgery. Some institutions allow patients to take their medicine with a small amount of apple sauce, which can be common in this patient population given the concern that the risk of a perioperative seizure can be more harmful to a patient than the small risk of aspiration.

Many patients with neuromodulation devices or presenting for device placement are also on a ketogenic diet, which consists of high fat, low carbohydrate, and moderate protein intake to cause ketosis. A ketogenic diet can reduce seizure frequency across various pediatric epilepsy and seizure syndromes [31,32,33]. Adherence to the continued ketotic state by limiting glucose-containing fluids and medications is important during the perioperative period for continued seizure suppression. Close collaboration with a pharmacy to coordinate non-glucose-containing fluids and medications as well as frequent glucose monitoring perioperatively is recommended by the authors [30,33].

## 5. Intraoperative Management for Device Placement

Given the uniqueness of pediatric physiology and pathophysiology, as well as the specialty training required for these specific procedures performed by neurosurgeons, these cases will likely be performed at pediatric tertiary or quaternary teaching hospitals by pediatric-trained neurosurgeons and pediatric-trained anesthesiologists. Therefore, pediatric anesthesiologists will have several considerations when providing anesthesia to this patient and surgical population.

Due to concurrent antiseizure medication use in this patient population and the metabolism of anesthetic agents via cytochrome p450 enzyme induction, many anesthetic medications require higher doses in the perioperative period. Higher doses of narcotics may be needed in response to noxious stimuli. Higher doses of neuromuscular blockade medications, such as rocuronium, may be needed to ensure adequate muscle relaxation throughout the operation. Conversely, antiseizure medications may act on GABAergic or other inhibitory mechanisms that produce a synergistic effect with propofol, therefore decreasing hypnotic anesthetic requirements [33]. Careful vigilance of potential responses to noxious stimuli as well as quantitative neuromuscular monitoring of the train-of-four ratio should be used.

Many anesthetic agents used in practice today can affect seizure threshold. Nitrous oxide has been shown to potentially provoke seizures in animals; however, this has not necessarily been shown in humans. Sevoflurane has been shown to create seizure-like activity at concentrations of 1.5–2 MAC in healthy adults [34], and intraoperative electrocorticograms have shown increased interictal spikes with 1.5 MAC sevoflurane in a case series of 13 adults with DRE [35]. Equivalent levels of sevoflurane are commonly used for inhalational induction in pediatric anesthesia. Although no definitive link has been established for this level of sevoflurane and clinical seizures, observational studies have shown increases in spike-and-wave discharges in epileptogenic regions with 1.5–2 MAC sevoflurane in pediatric patients undergoing intraoperative electrocorticography [36,37]. On the other hand, isoflurane has shown anti-convulsant properties and has been used in an intensive care setting to treat patients with severe refractory status epilepticus [33,38]. Meperidine, an opioid commonly used perioperatively to treat shivering from anesthesia, has concern for myoclonus and tonic–clonic seizure activity. The FDA drug label for meperidine warns that it may increase the risk of seizure in patients with or without a pre-existing seizure disorder, and that prolonged use may do so due to accumulation of its metabolite normeperidine [39]. Benzodiazepines are used to treat seizures and are commonly used in the perioperative period. Providing benzodiazepines as anxiolysis at the beginning of a case can potentially prevent a seizure from occurring intraoperatively. Intravenous anesthetic agents such as barbiturates and ketamine are used clinically as anticonvulsants, but also seem to be excitatory at lower doses in animal studies [40,41].

## 6. Device-Specific Perioperative Considerations

Each neuromodulation device also introduces unique intraoperative concerns. Although there is no standardized pediatric perioperative workflow for implanted neuromodulating devices during non-neurologic surgery, published adult guidance and expert consensus support can provide insights for the pediatric population. Devices are updated frequently and the most current recommendations can often be found with the device company itself. The device representative or most current information from device manufacturers should be consulted if there is any uncertainty regarding best practice for the specific device for a patient in their perioperative experience. Past neuromodulation-related adverse events such as bradycardia, apneas, and chronic cough should also be assessed perioperatively.

### 6.1. Vagus Nerve Stimulation Considerations

VNS placement can have important cardiovascular complications. Bradycardia is mediated through direct parasympathetic stimulation of cardiac vagal efferent fibers acting on the atria, the sinoatrial node, the atrioventricular node, and the ventricular conduction system [42]. Pediatric bradyarrhythmias have been described with VNS stimulation. Rarely, lead impedance testing can cause bradyarrhythmias, and there are rare case reports of bradyarrthymias correlating with stimulation intervals [10,43].

The most common side effect of VNS therapy is obstructive sleep apnea (OSA). The mechanism is hypothesized to be due to VNS-induced left vocal cord adduction during stimulation leading to upper airway obstruction [44,45]. There is an increased incidence of diminished airway patency and sleep-disordered breathing in pediatric patients with VNS, most commonly with children with no previous OSA symptoms to new mild-to-moderate OSA. The greatest decrement in laryngopharyngeal function occurs within the first year after VNS implantation [46]. Thus, screening for OSA symptoms before and after VNS implantation is recommended. The OSA symptoms are commonly managed by adjusting nighttime device parameters.

The most common complication of VNS placement is infection, and children ages 4–11 years may be at the greatest risk compared with older children [47]. Children, especially those with cognitive delay who are more likely to manipulate the surgical site, are at higher risk of wound infection that may necessitate explantation [48]. Children also require repeat surgeries for battery replacements over their lifetime and repeat surgeries may also increase infection risk. A retrospective review of pediatric patients undergoing VNS surgery showed that the incident rate of infection was 19 times greater in patients who had at least two prior revisions compared to first-time revisions [49]. A 2021 multi-institutional retrospective review found the most common pathogen for VNS infection in a mixed pediatric and adult population was methicillin-sensitive staphylococcus [50]. Antibiotics with or without generator removal may be sufficient to clear the infection. In other cases, the generator and the leads must both be removed. Surgical infection prophylaxis targets skin flora (usually cefazolin if no beta-lactam allergy) and the exact regimen depends on local hospital infection guidelines. Higher local rates of methicillin-resistant staphylococcus raise consideration for the addition of a second antibiotic such as vancomycin. Many institutions also adopt pre-operative chlorhexidine washes to further reduce infection risk. Often, there is no standardized protocol for post-operative antibiotics for surgical site infection prophylaxis in VNS surgery. In the Clinical Practice Guidelines for Antimicrobial Prophylaxis in Surgery published by The American Society of Health-System Pharmacists, they recommend less than 24 h of antimicrobial prophylaxis for most procedures (their pediatric recommendations are based on expert opinion) [51]. A case series of pediatric patients at a single center compared infection rates between two surgeons for VNS placement with standard preoperative antibiotics, but with one surgeon prescribing an additional week of postoperative antibiotics. There was no difference in infection rates despite the extended treatment with postoperative antibiotics [52].

After implantation, these children often return for anesthesia services for neuromodulator-related surgery or non-neuromodulator-related procedures and imaging. Because the updated VNS design involves a closed-loop system that senses an increase in heart rate to initiate therapy, it is important to turn off the VNS prior to surgery or imaging procedures when tachycardia may be a common event. Currently, VNS devices for pediatric epilepsy are manufactured by LivaNova (VNS Therapy^®^). The implantable pulse generator battery usually lasts 3–8 years [53]. Battery replacement is a minimally invasive surgery but unlike adults, children generally do not tolerate the procedure under light sedation and require general anesthesia. Once the battery is replaced, the device can be activated in the operating room or at a later time.

Electrocautery can also damage the device and/or affect the stimulation. Bipolar cautery is preferred over monopolar cautery due to the risk of arcing current on the device [53]. If the VNS device is in active mode for surgery, patients may have their own personal magnet placed over the internal pulse generator in the operating room by the anesthesiologist to turn off the device if needed. Alternatively, if the VNS magnet is unavailable, any typical pacemaker magnet (with a strength > 50 gauss) can also deactivate the VNS [54]. If monopolar cautery is used, the grounding pad should be placed as far away from the generator as possible. Immediate postoperative management for non-neurologic surgeries includes prompt reactivation with interrogation to confirm proper function. This requires coordination with the pediatric neurology team. Close monitoring is necessary for stimulation-related respiratory events such as apnea or stridor. Emergence delirium or agitation may mimic seizures, making preoperative knowledge of seizure semiology essential in postoperative assessment.

Central line placement, specifically in the internal jugular or subclavian veins, requires special consideration in a patient with a VNS device. VNS leads most commonly run from the generator in the left chest, up the left neck, and around the left vagus nerve [55]. Therefore, left-sided central lines in that area should be avoided. Ultrasound may be used to identify the hyperechoic cylindrical leads in the cervical region. Caution should be used to minimize neck rotation and traction on the leads when placing lines in the neck and upper chest. Peripherally inserted central catheters (PICC) or femoral lines are alternatives that can be used if central access is needed.

Extra care should be taken when administering neuraxial anesthesia to a child with an implanted VNS device. Neuraxial anesthesia can produce a sympathetic blockade corresponding to the dermatomal level at which the block rises. If the sympathetic cardiac accelerator fibers (T1–T4) are affected, there may be unopposed parasympathetic tone resulting in significant bradycardia. If neuraxial anesthesia is planned, it is even more prudent to turn off VNS stimulation during surgery and take extra caution when turning the stimulation back on postoperatively due to the potential synergistic bradycardia effects. Patients may benefit from continuous electrocardiography monitoring and having atropine immediately available [56,57].

For radiology studies, VNS devices are MRI 1.5 T conditional [53]. VNS devices are also 3 T conditional but preclude scanning where the device is located between the C7 and T8 levels of the spine. Prior to the MRI, the VNS stimulation therapy must be turned off (all output currents set to 0.0 mA) [47].

### 6.2. Deep Brain Stimulation Considerations

DBS devices are placed intracranially and have risks associated with intracranial surgery, such as bleeding. A multicenter pediatric DBS registry, which includes indications for other neurodevelopmental disorders, reported infection rates comparable to adult studies [58]. Interestingly, infection rates for patients with movement disorders were higher than for patients with DRE. To minimize damage to the device, companies recommend turning the DBS device off for surgical procedures, if possible. Unfortunately, there is limited safety data on the effects of electrocautery on DBS. Monopolar electrocautery has been used safely, but bipolar electrocautery is recommended due to the theoretical decreased risk from the reduced electromagnetic field [53]. The device should be re-activated postoperatively and interrogated to confirm proper functioning.

DBS devices have intracranial leads connected to a subcutaneous subclavicular pulse generator. The leads descend from the scalp to a retroauricular path down the neck to the chest or abdomen generator [59]. Central line placement in the neck or chest also requires special consideration. The tunneled DBS leads commonly run subcutaneously along the lateral neck so the contralateral side should therefore be chosen for a central line. Caution should be used to minimize neck rotation and traction on the leads.

For radiology studies, DBS are 1.5 T conditional and newer models are 3 T conditional, and may even have an “MRI mode” to help protect the device from overheating during the scan. Without the “MRI mode,” manufacturers recommend turning the device off and reactivating afterwards with interrogation to confirm proper functioning [53].

### 6.3. Responsive Neurostimulation Considerations

RNS devices are implanted within the cranium, usually within the nondominant parietal region without subcutaneous extension of leads or a separate chest wall generator. There are risks associated with intracranial surgery, such as bleeding. RNS devices may also require intraoperative electrocorticography to map the precise location of the seizure, which requires an anesthetic that minimizes electrical suppression. As young children are generally unable to tolerate awake craniotomy, techniques used include low-dose sevoflurane, propofol-based, and dexmedetomidine- or remifentanil-based anesthetics.

Like DBS, device manufacturers recommend turning off RNS stimulation, if possible, prior to surgery. Bipolar electrocautery is recommended >2 cm from the device because cautery can damage it. After reactivation, the RNS device should be interrogated to insure proper functioning. For radiology studies, RNS devices are MRI 1.5 T conditional and have MRI modes that should be activated during scanning to prevent leads from overheating [53].

## 7. Future Directions

It is estimated that there are 374.8 cases of pediatric epilepsy per 100,000 children in the peak age group of 5–9 years old [3]. There were 18.15 million cases of epilepsy in children worldwide in 2021 [60], yet there is a lack of pediatric data for VNS, DBS, and RNS devices. VNS therapy remains the most well-studied neuromodulation modality in pediatric patients. VNS is the only FDA-approved device for pediatric epilepsy, while DBS has pediatric FDA approval for dystonia. The lack of robust pediatric data on these devices means that much of our knowledge is extrapolated from either adult data or from other indications for these devices in pediatric patients. There have been a handful of studies reported in the literature, with 60 pediatric patients enrolled in the original compassionate use protocol, subsequent multiple small, prospective and retrospective VNS studies [11,12], and a large, multicenter, retrospective European study of 347 children who underwent VNS implantation [14]. Smaller still are the populations of pediatric patients with DBS and RNS devices. There was a systematic review of 72 pediatric DBS patients and 46 pediatric RNS patients [18], and 88 pediatric patients in a meta-analysis of DBS and RNS [20]. These are a proportionally small number considering the worldwide burden of pediatric epilepsy. The safety profile of VNS, DBS, and RNS are similar in pediatrics as in adult patients, but key differences remain to be seen. Long-term outcomes are particularly pertinent as children have longer time periods with the implanted device. Device longevity and hardware complications will be key long-term outcomes. Infection risk is one of the main concerns for pediatric patients due to high physical activity and decreased discipline to avoid manipulation of fresh surgical areas. Furthermore, neurodevelopment and linear growth in children may cause lead migration and dislodgement, possibly requiring revisions over the long-term treatment course. There is a need for development of multicenter pediatric registries to follow up on children as they progress through childhood, adolescence, and adulthood with their neuromodulation devices, integrating neurodevelopmental and long-term device outcomes as primary endpoints.

As more children receive these devices, standardized perioperative protocols and checklists will be needed for centers in their perioperative and imaging workflows. Continuous training and education for physicians and staff will help build perioperative teams that have familiarity with neuromodulation devices. Finally, with greater advocacy for device trials and regulatory support for these devices to gain FDA approval in pediatric populations, there will be fertile ground for developing smaller-sized leads and generators for pediatric patients, improved MRI compatibility, and closed-loop algorithms specific to the developing pediatric brain.

## 8. Search Strategy

A search was completed in PubMed and OpenEvidence using the key terms: “drug-resistant epilepsy,” “pediatric epilepsy,” “vagus nerve stimulation,” “deep brain stimulation,” “responsive neurostimulation,” and “perioperative considerations for neuromodulation devices” in January 2026. The search included meta-analysis, randomized controlled trials, case reports, clinical trials, observational studies, and reviews in the last 30 years with a focus on literature published within the last 5 years.

## Figures and Tables

**Table 1:** Neuromodulation devices used in pediatric epilepsy.

	Vagus Nerve Stimulation (VNS)	Deep Brain Stimulation (DBS)	Responsive Neurostimulation (RNS)
Manufacturer	LivaNova (VNS Therapy^®^)	Medtronic	NeuroPace
FDA pediatric approval	≥4 years old	≥7 years old for dystonia, but off-label pediatric use for epilepsy [9]	Off-label pediatric use
Implant Location	Typically left cervical vagus nerve; generator in chest	Intracranial leads; generator in upper chest or abdomen	Intracranial leads; skull-mounted generator
Magnet interaction	Yes—can suspend or activate stimulation	No	No
Perioperative concerns	Bradycardia, bronchospasm, decreased laryngopharyngeal patency, electromagnetic interference	Electromagnetic interference, lead heating, neurological effects	Electromagnetic interference, loss of sensing, data corruption
Preoperative recommendation	Turn off stimulation (including Magnet and AutoStim modes)	Ideally turn off stimulation	Ideally turn off stimulation
Intraoperative recommendation	Electrocautery should be avoided near the device Avoid left-sided central line placement (VNS is often on the left) Potential for exaggerated bradycardia with neuraxial anesthesia	Bipolar electrocautery recommended Consider placing central line contralateral to device	Avoid monopolar electrocautery; if >2 cm from the device, then can consider bipolar electrocautery No concerns for central line placement
Postoperative reactivation	Magnet or programmer; interrogate device afterwards	Programmer; interrogate device afterwards	Programmer; interrogate device afterwards
MRI concerns	1.5 T conditional, 3 T conditional except for scanning between C7 and T8 Stimulation must be off	1.5 T conditional; some models 3 T conditional; some have MRI mode	1.5 T conditional; has MRI mode to inactivate stimulation and protect leads from overheating

## Data Availability

No new data were created or analyzed in this study.

## Abbreviations

The following abbreviations are used in this manuscript:

DBS	Deep brain stimulation
DRE	Drug-resistant epilepsy
EEG	Electroencephalography
MRI	Magnetic resonance imaging
PICC	Peripherally inserted central catheter
OSA	Obstructive sleep apnea
RNS	Responsive neurostimulation
VNS	Vagus nerve stimulation
